# Vitamin D insufficiency and its association with adipokines and atherogenic indices in patients with metabolic syndrome: A case-control study

**DOI:** 10.3389/fendo.2023.1080138

**Published:** 2023-01-20

**Authors:** Farshad Amirkhizi, Zeinab Khademi, Soudabeh Hamedi−Shahraki, Mehran Rahimlou

**Affiliations:** ^1^ Department of Nutrition, Faculty of Public Health, Zabol University of Medical Sciences, Zabol, Iran; ^2^ Department of Public Health, Sirjan School of Medical Sciences, Sirjan, Iran; ^3^ Department of Epidemiology and Biostatistics, Faculty of Public Health, Zabol University of Medical Sciences, Zabol, Iran; ^4^ Department of Nutrition, Faculty of Medicine, Zanjan University of Medical Sciences, Zanjan, Iran

**Keywords:** vitamin D, metabolic syndrome, cardio metabolic, adipokines, cardiovacsular disease(s)

## Abstract

**Introduction:**

Vitamin D deficiency is one of the most common nutritional disorders in most countries of the world. The present study was designed and implemented with the aim of investigating the relationship between vitamin D deficiency and the level of adipokines, atherogenesis indicators and factors related to metabolic syndrome.

**Methods:**

This case-control study was done on 195 patients with metabolic syndrome aged 20-50 y who attended the health centers in Zabol County, northeast Iran, between April 2021 and January 2022. Anthropometric and biochemical parameters were measured for all subjects with standard methods. To determine serum 25(OH)D levels, we used enzymatic linked immunosorbent assay (ELISA) kits. Atherogenic index of plasma (AIP) was calculated as log (TG/HDL-c). The visceral adiposity index (VAI) and the lipid accumulation product (LAP) were estimated according to standard formulas.

**Results and Discussion:**

Participants in the case group had lower serum levels of 25(OH)D compared to controls (19.8 ± 6.2 ng/ml vs. 41.2 ± 9.7ng/ml, P<0.001). We found that the mean serum levels of fasting blood sugar (P=0.023) and TG (P=0.008) as well as HOMA-IR (P=0.023) were significantly higher in the cases compared to controls. Also, patients with MetS and vitamin D insufficiency (cases) had higher AIP (P=0.040) and LAP (P=0.012) than controls. Furthermore, serum 25(OH)D levels showed significant inverse correlations with serum RBP-4 and a positive correlation with serum omentin-1 concentrations. The results of the present study showed that vitamin D deficiency correlated with some of the cardiometabolic risk factors among the patients with MetS.

## Introduction

Several studies have shown a rise in the percentage of overweight and obese adults in recent decades. According to the world health organization (WHO) reports, 39% of adults aged 18 years and over were overweight in 2016, and 13% were obese. Also, it has been reported that 39 million children under the age of 5 were overweight or obese in 2020 (1).

Adults, teenagers, and children are all affected by the issue (2–4). Numerous systemic illnesses, such as metabolic syndrome (MetS), type 2 diabetic mellitus (T2DM), atherosclerosis, cardiovascular problems, and cancers, are caused on by obesity (5). Currently, the prevalence of T2DM is rising, and by 2040, it has been estimated that over than 642 million persons (10% of the population) will have this disease. People who have low levels of serum 25-hydroxyvitamin D3 [25(OH) D3] have a considerably greater risk of developing T2DM and MetS (6, 7). Vitamin D deficiency have been linked to a higher odds ratio of MetS and T2DM, according to some of cohort studies (8, 9). It has been reported that vitamin D receptor (VDR) changes involved in the pathogenesis of some chronic disorders such as diabetes (10), autoimmune diseases (11), nonalcoholic liver disease (12), cardiovascular disease(CVD) (13), and cancer (14).

Individuals with risk factors for CVD frequently have low serum 25(OH)D3 concentration. It has been reported that there was a significant inverse association between serum levels of 25(OH)D3 and some cardiometabolic risk factors such as fasting blood sugar (FBS), hemoglobin A1c (HbA1c), total cholesterol (TC), triglyceride (TG), body mass index (BMI), waist circumference (WC), and atherogenic indices (Castelli Risk Index I (CRI I), Castelli Risk Index II (CRI II), and Atherogenic index of plasma (AIP) (15).

Obesity, especially central obesity, is one of the most important risk factors for T2DM, which causes insulin resistance and inflammation due to the increase of fat tissue in the body, which also increases the risk of CVD (16). The mechanisms involved in the relationship between obesity, adipose tissue dysfunction and MetS have not yet been precisely identified (17). It has been reported in some pervious research that adipose tissue metabolic alterations, such as dysregulated adipokine production, may mediate the association between various obesity phenotypes and vulnerability to some chronic disorders such as MetS (18). Adipokines have long been recognized as significant hunger and satiety moderators as well as critical regulators of energy homeostasis, inflammation, immunological function, blood pressure, vascular function, insulin levels, and glucose and lipid metabolism (19).

In recent years, some studies have shown that there is a bidirectional relationship between vitamin D and obesity (20, 21). In obese people, due to the higher fat mass, high amounts of vitamin D are trapped in fat tissues, and therefore, these people may need higher amounts of vitamin D. On the other hand, some findings have revealed that vitamin D supplementation may have a positive effect on obesity prevention and treatment (22, 23). In addition, some researchers have suggested that some effects of vitamin D deficiency in the etiology of obesity and T2DM are exerted through disturbances in the concentration of adipokines such as leptin and adiponectin (22, 24).

Therefore, considering the importance of vitamin D in the prevention and treatment of metabolic syndrome and the existence of conflicting evidence regarding the effective mechanisms in the results observed in previous studies, the present study designed to investigate the relationship between vitamin D deficiency with adipokines and atherogenic indices in patients with metabolic syndrome.

## Material and methods

### Study participants

This case-control study was done on 195 patients with Mets aged 20-50 y who attended the health centers in Zabol County, southeast Iran, between April 2021 and January 2022. Serum omentin-1 levels as a key variable obtained from Zorlu et al. study (25), was used to estimate the sample size. Considering the study power of 80%, a type I error of 5%, and ratio of controls to cases as 2, we required 65 cases and 130 controls for this study.

Cases were MetS patients with vitamin D insufficiency and controls were MetS patients with optimal vitamin D status. Controls were frequency-matched with cases on age ( ± 2 years), sex, and BMI ( ± 1 kg/m^2^) and were selected from patients visiting the same health centers. Vitamin D insufficiency was defined, according to the previous studies and Endocrine Society clinical practice guidelines (26, 27), as serum 25(OH)D levels < 30 ng/ml and those who had serum 25(OH)D levels ≥ 30 ng/ml were considered sufficient.

MetS was diagnosed based on the National Cholesterol Education Program-Adult Treatment Panel III (NCEP-ATP III) consensus (28) as the presence of at least three of the following criteria: (1) a waist circumference (WC) > 102 cm in males and > 88 cm in females, (2) a fasting blood sugar (FBS) ≥ 100 mg/dl, (3) a high serum triglyceride (≥150 mg/dl), (4) a low serum HDL-c (< 40 mg/dl in males and < 50 mg/dl in females), (5) a high blood pressure (SBP ≥ 130 mmHg or DBP ≥ 80 mmHg).

In this study, individuals with a prior history of cardiovascular diseases, endocrine disorders such as diabetes and hypo-/hyperthyroidism, cancer, renal or liver dysfunction were excluded. The individuals were also excluded if taking antioxidant supplements like selenium, carotenoids, and vitamins E and C, as well as any medications known to induce metabolic or hormonal changes such as estrogens, corticosteroid drugs and lipid-lowering medications within three months before enrollment the study. Following a specific diet, using fish oil supplement and taking anti-inflammatory medications in the past three months were among the exclusion criteria as well. Eligible individuals, including 65 cases and 130 controls were recruited for the study.

All study participants signed an informed consent form after explaining the aims and the study methodology. The study protocol was approved by the Ethics Committee of Zabol University of Medical Sciences (Approval code: IR.ZBMU.REC.1399.156).

### Assessment of anthropometric variables, blood pressure and physical activity

Anthropometric parameters were measured for all subjects. Height was measured without shoes in an upright position using a fixed non-stretchable tape with a precision of 0.1 cm. Weight was measured on light clothing by a Seca scale to the nearest 0.1 kg. Body mass index (BMI) was calculated as weight (kg) divided by squared height (m^2^). Finally, the waist circumference (WC) was measured between the lower rib margin and the iliac crest at the end of a normal expiration. A bioelectrical impedance analysis (BIA) system (InBody S10, JMW140, Korea) was applied to measure the percentage of body fat mass (%FM) and visceral fat level (%VF).

Blood pressure was measured in a sitting position, after a 15-min rest, using a mercury sphygmomanometer twice with 5-min intervals. The mean of two measurements was considered as the final systolic (SBP) and diastolic (DBP) blood pressure.

To evaluate the physical activity levels of the participants, we applied a short form of International physical activity questionnaire (IPAQ) and then classified into 3 categories of “low,” “moderate,” and “vigorous” activity according to the IPAQ scoring guideline (29).

### Laboratory measurements

Blood samples were collected from all patients after almost 12 h of overnight fasting and centrifuged at 3500 rpm for 10 min to separate the sera. Then, the separated sera were immediately distributed in aliquots and stored at -70^○^C until analysis.

Fasting blood sugar (FBS) as well as serum levels of total cholesterol (TC), high-density lipoprotein cholesterol (HDL-c), and low-density lipoprotein cholesterol (LDL-c) were determined using the standard enzymatic-colorimetric method on an automatic biochemical Hitachi 717 analyzer (Hitachi, Boehringer Mannheim, Japan) through commercial kits (Pars-Azmoon Co., Tehran, Iran) with inter- and intra-assay coefficient variances (CVs) of lower than 5%. Non-HDL-cholesterol was calculated as the TC minus HDL-c. Serum insulin levels were determined based on the radioimmunoassay method using the commercial kit (Immunotech, Prague, Czech Republic), which is sensitive to 0.5 mU/ml variations in serum levels of insulin and its intra- and inter-assay coefficients of variation were 3.8% and 6.2%, respectively. Insulin resistance was estimated with the homeostasis model assessment method (HOMA-IR) using the suggested equation of Matthews et al. (30): HOMA-IR= [fasting insulin (U/l) × fasting glucose (mg/dl)]/405.

Atherogenic index of plasma (AIP) was calculated as log (TG/HDL-c) (31). The visceral adiposity index (VAI) and the lipid accumulation product (LAP) were estimated according to suggested formulas for each gender as follows:


$$\begin{array}{l} \text{Males: VAI= }\left[\text{WC/}\left(39.68 + 1.88 \times BMI\right)\right]\text{ × }\\ \left(TG/1.03\right)\text{ × }\left(1.31/HDL-c\right) \end{array}$$

(32)


$$\begin{array}{l} \text{Females: VAI= }\left[\text{WC/}\left(36.58 + 1.89 \times BMI\right)\right]\text{ × }\\ \left(TG/0.81\right)\text{ × }\left(1.52/HDL-c\right) \end{array}$$

Males: LAP= [WC (cm) -65] × [TG (mmol/l)]

(33)


Females: LAP= [WC (cm) -58] × [TG (mmol/l)]

To determine serum 25(OH)D levels, we used enzymatic linked immunosorbent assay (ELISA) kits (DIAsource Immunoassays SA, Louvain-laNeuve, Belgium) according to the manufacturer’s instructions. Serum levels of omentin-1, chemerin, vaspin, and retinol binding protein 4 (RBP-4) were measured using the Human ELISA kits (Shanghai Crystal Day Biotech Co., Ltd.) according to the manufacturer’s instructions. The intra- and inter-assay CV of adipokines was lower than 4.7% and 7.8%, respectively.

### Statistical analysis

The results were presented as mean ± standard deviation for normally distributed continuous data and frequency (percent) for categorical data. The non-normally distributed data were expressed as the median and interquartile range (IQR). General characteristics were compared between cases and controls using an independent samples t-test and Pearson chi-squared test, as appropriate. In addition, between-groups differences in normally and non-normally distributed cardiometabolic parameters and adipokines were investigated using independent samples t-test and non-parametric Mann–Whitney U-test, where appropriate. To examine the association between serum levels of 25(OH)D with adipokines and cardiometabolic parameters, multiple linear regression was applied by adjusting for age, sex, smoking, physical activity level, and BMI. Data were analyzed using IBM SPSS version 25 (IBM Corp., Armonk, NY, USA) and statistical significance was considered as a p-value< 0.05.

## Results

### Characteristics of cases and controls

The study participants consisted of 195 patients with MetS (65 cases and 130 controls). The mean age of cases and controls was 38.0 ± 5.5 and 37.5 ± 5.6 y, respectively that was non-significant. Totally, 34% of patients participating in this study were male and 66% were female. No significant differences were found between cases and controls with respect to age, BMI, WC, fat mass, and visceral fat (**Table 1**) (P>0.05). In addition, the distribution of participants with regard to sex, smoking status, PAL, and education level was not significantly different between the study groups (P>0.05). As shown in **Table 1**, participants in the case group had lower serum levels of 25(OH)D compared to controls (19.8 ± 6.2 ng/ml vs. 41.2 ± 9.7*ng/ml*, P<0.001).

**Table 1:** General characteristics of cases and controls.

Variables	Cases (n=65)	Controls (n=130)	*P*-value
Male, *n* (%)	21 (32.3)	45 (34.6)	0.748^†^
Age (years)	38.0 ± 5.5	37.5 ± 5.6	0.556^‡^
Weight (kg)	89.7 ± 11.6	90.5 ± 12.1	0.673^‡^
BMI (kg/m^2^)	32.0 ± 2.1	31.6 ± 1.9	0.289^‡^
WC (cm)	96.7 ± 8.0	94.7 ± 9.4	0.141^‡^
Fat mass (%)	44.3 ± 7.2	42.8 ± 7.3	0.185^‡^
Visceral fat (%)	13.2 ± 4.1	12.8 ± 4.0	0.534^‡^
PAL, *n* (%)			
Low	47 (72.3)	90 (69.2)	0.904^†^
Moderate	15 (23.1)	33 (25.4)	
Vigorous	3 (4.9)	7 (5.4)	
Smokers, *n* (%)	14 (21.5)	31 (23.8)	0.718^†^
Education level, *n* (%)			
Primary school	24 (36.9)	49 (37.7)	0.776^†^
Secondary & high school	26 (40.0)	46 (35.4)	
Diploma & university	15 (23.1)	35 (26.9)	
25(OH)D (ng/ml)	19.8 ± 6.2	41.2 ± 9.7	<0.001^‡^

BMI, body mass index; WC, waist circumference; PAL, physical activity level.

Values are reported as mean ± standard deviation for continuous variables and number (%) for categorical variables.

P-values<0.05 were considered significant.

^†^Pearson chi-square test.

^‡^Independent samples t-test.

### Cardiometabolic parameters in cases and controls

**Table 2** shows the differences in the cardiometabolic parameters between the cases and controls.

**Table 2:** Comparison of cardiometabolic parameters between cases and controls.

Variables	Cases (n=65)	Controls (n=130)	*P*-value^†^
FBS (mg/dL)	106.1 ± 13.6	101.9 ± 11.1	0.023
Insulin (µU/mL)	18.1 ± 5.8	16.8 ± 5.2	0.116
HOMA-IR	4.8 ± 2.1	4.2 ± 1.5	0.032
TG (mg/dL)	190.0 ± 27.7	180.0 ± 23.1	0.008
TC (mg/dL)	197.5 ± 23.1	191.8 ± 23.9	0.113
LDL-c (mg/dL)	137.3 ± 27.2	132.5 ± 23.4	0.201
HDL-c (mg/dL)	46.7 ± 5.8	47.1 ± 6.4	0.719
non-HDLC	150.8 ± 23.3	144.8 ± 24.3	0.101
AIP	0.61 ± 0.08	0.58 ± 0.08	0.040
LAP	78.25 ± 21.4	69.79 ± 22.20	0.012
VAI	0.99 ± 0.08	0.97 ± 0.09	0.239
SBP (mmHg)	127.2 ± 3.8	126.5 ± 4.1	0.304
DBP (mmHg)	83.7 ± 3.2	82.9 ± 2.7	0.083

FBS, fasting blood sugar; HOMA-IR, homeostasis model assessment of insulin resistance; TG, triglyceride; TC, total cholesterol; LDL-c, low-density lipoprotein cholesterol; HDL-c, high-density lipoprotein cholesterol; AIP, atherogenic index of plasma; LAP, lipid accumulation product; VAI, visceral adiposity index; SBP, systolic blood pressure; DBP, diastolic blood pressure.

Values are reported as mean ± standard deviation.

P-values<0.05 were considered significant.

^†^Independent samples t-test.

The mean serum levels of FBS (*P*=0.023) and TG (*P*=0.008) as well as HOMA-IR (*P*=0.023) were significantly higher in the cases compared to controls. Furthermore, patients with MetS and vitamin D insufficiency (cases) had higher AIP (*P*=0.040) and LAP (*P*=0.012) than controls, whereas, there were no significant differences in serum levels of insulin, TC, LDL-c, HDL-c, and non-HDL as well as VAI, SBP, and DBP.

### Serum adipokines in cases and controls

**Figure 1:**
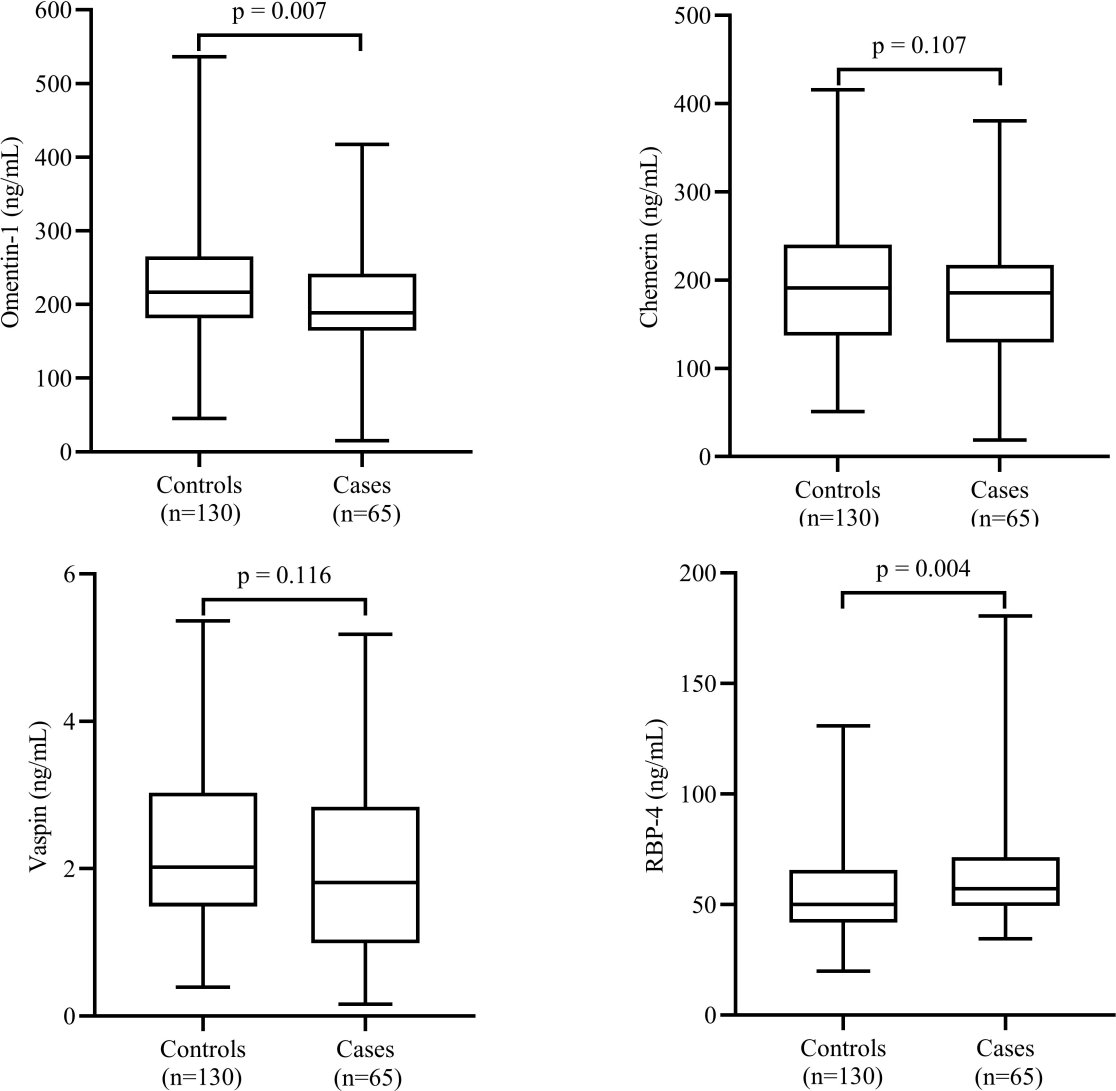
Comparison of serum levels of adipokines between cases and controls. Data are shown as median (interquartile range). P-values obtained from Mann–Whitney U test. P-values < 0.05 were considered significant.

The comparisons of the serum levels of adipokines between the cases and controls are shown in **Figure 1**. The mean serum levels of omentin-1 were significantly higher (*P*=0.007) and serum levels of RBP-4 (*P*=0.007) were significantly lower in the cases compared to controls. However, we failed to find any significant difference in mean serum levels of vaspin and chemerin between the two groups.

### Association of serum vitamin D with serum adipokines and cardiometabolic parameters

The results of multiple linear regression models, which investigated the associations between serum 25(OH)D levels with serum adipokines and cardiometabolic parameters are reported in **Table 3**. Serum 25(OH)D levels showed significant inverse correlations with serum RBP-4 (*β*=-0.305, *P*<0.001), FBS (*β*=-0.189, *P*=0.007), insulin (*β*=-0.170, *P*=0.017), HOMA-IR (*β*=-0.206, *P*=0.004), TG (*β*=-0.431, *P*<0.001) as well as AIP (*β*=-0.360, *P*<0.001) and LAP (*β*=-0.281, *P*<0.001) after adjustment for potential confounders including age, sex, PAL, and BMI. There were significant positive correlations between serum 25(OH)D levels and serum levels of omentin-1 (*β*=0.197, *P*=0.007). When regression analyses were performed by adjustment for potential confounders, serum 25(OH)D levels did not show any significant correlations with serum chemerin, vaspin, TC, LDL-c, HDL-c, non-HDL, SBP, DBP as well as VAI.

**Table 3:** Multiple linear regression analysis for the association between serum 25(OH)D concentrations with adipokines and cardiometabolic parameters (*n*=195).

Model	B	S.E.	β	*P*-value^†^
Omentin-1 (ng/mL)	1.028	0.376	0.197	0.007
Chemerin (ng/mL)	0.433	0.341	0.094	0.205
Vaspin (ng/mL)	0.010	0.006	0.118	0.110
RBP-4 (ng/mL)	-0.476	0.110	-0.305	<0.001
FBS (mg/dL)	-0.172	0.063	-0.189	0.007
Insulin (µU/mL)	-0.071	0.030	-0.176	0.017
HOMA-IR	-0.027	0.009	-0.206	0.004
TG (mg/dL)	-0.811	0.125	-0.431	<0.001
TC (mg/dL)	-0.188	0.130	-0.106	0.150
LDL-c (mg/dL)	-0.158	0.136	-0.085	0.248
HDL-c (mg/dL)	-0.037	0.033	-0.081	0.266
non-HDLC	-0.225	0.132	-0.125	0.089
AIP	-0.003	0.001	-0.360	<0.001
LAP	-0.468	0.109	-0.281	<0.001
VAI	-0.002	0.001	-0.116	0.115
SBP (mmHg)	-0.033	0.022	-0.110	0.134
DBP (mmHg)	-0.019	0.016	-0.089	0.223

RBP-4, retinol binding protein 4; FBS, fasting blood sugar; HOMA-IR, homeostasis model assessment of insulin resistance; TG, triglyceride; TC, total cholesterol; LDL-c, low-density lipoprotein cholesterol; HDL-c, high-density lipoprotein cholesterol; AIP, atherogenic index of plasma; LAP, lipid accumulation product; VAI, visceral adiposity index; SBP, systolic blood pressure; DBP, diastolic blood pressure; B, unstandardized coefficient; S.E., standard error.

^†^Adjusted for age, sex, smoking, physical activity level, and BMI.

P-values<0.05 were considered significant.

## Discussion

The results of the present study were showed that participants with MetS and vitamin D insufficiency had higher levels of some risk factors for CVD. Also, we found an inverse association between serum levels of 25(OH)D and some CVD related biomarkers.

Vitamin D insufficiency is currently a major global health issue due to its high incidence (34). According to the results of a meta-analysis study, the prevalence of vitamin D deficiency in the Iranian population is 56% (64% in women and 44% in men) (35). Given that the majority of body cells have vitamin D receptors and para/autocrine vitamin D metabolic activity, vitamin D effects extend beyond the regulation of bone tissue (36, 37). The potential therapeutic benefits of vitamin D3 can be obtained by keeping vitamin levels in adults in the range of 30-80 ng/mL (75-200 nm/L), according to expert recommendations that published in 2018 (38). We found that the serum levels of FBS, TG and HOMAIR in the participants with vitamin D deficiency were significantly higher than control group. Also, we found a significant inverse association between 25(OH)D levels with serum levels of FBS, TG and HOMAIR. In line with our findings, Schmitt et al. were reported that in postmenopausal women, vitamin D deficiency was correlated with higher prevalence of MetS, as well as hypertriglyceridemia (39). In other study, Lee et al. found that lower serum levels of 25(OH)D was associated with higher waist circumference, TG and insulin resistance (40). Likewise, Zhu and Heil reported that 25(OH)D levels were inadequate in 50% of the study population, formed by residents of Shanghai, China, aged 19–70 years and were associated with the presence of MetS, and they found that higher serum levels of vitamin D was associated with lower LDL and total cholesterol concentration (41). Increasing evidence points to a strong association between vitamin D insufficiency and a decrease in insulin secretion in both people and animal models (42). Numerous studies have suggested that reduced levels of vitamin D disrupt insulin sensitivity, beta-cell function, or both to cause the onset of T2DM and insulin resistance (43–46). On the other hand, some studies evaluated the effect of vitamin D supplementation in people suffering from vitamin D deficiency and its effect on factors related to metabolic syndrome. In research that was conducted among the Asian women with insulin resistance and baseline 25(OH)D levels below 20 ng/mL, it has been reported that vitamin D supplementation with dose of 4,000 IU led to a significant improvement in the insulin sensitivity (47). Also, in a systematic review and meta-analysis study on 19 randomized controlled trials (RCT), it has been reported that compared with the control group, the short-term vitamin D supplementation group had a significant reduction in HbA1c, insulin resistance, and insulin concentration (48). The mechanisms of vitamin D reducing the risk of T2D include, improved insulin sensitivity, and reduced insulin resistance (49–51). It has also been shown that vitamin D reduces inflammation as one of the main factors involved in the pathogenesis of T2DM due the dysfunction in the insulin sensitivity and pancreatic beta-cell function (52–54).

In the present study, we found that participants in the vitamin D deficiency group had higher AIP and LAP than control group. Wang et al. in a population-based study on 1475 participants showed that serum 25(OH)D concentration was inversely correlated with TG, LDL-C and AIP (55). In other study, Mahmoodi et al. in a case control study among the participants with T2DM found that all of the atherogenic indices including AIP, CRII, and Atherogenic Coefficient (AC) significantly reduced with improved serum vitamin D status (56). It has been reported in several studies that vitamin D deficiency increase risk of dyslipidemia (57, 58). According to results from the Karhapää et al. investigation, in middle-aged Finnish men, serum 25(OH)D levels are inversely related to TC, TG, and LDL-C (59). The results of a cross-sectional study on the Danish population showed that a 10-unit increase in vitamin D concentration was associated with a significant decrease in the level of dyslipidemia-related factors (60). AIP value, which is obtained by applying a logarithmic transformation to the result of dividing the plasma TG value by the plasma HDL value, has recently been discovered to be a reliable indicator of the risk of atherosclerosis and CVD (61). In our study, AIP value in the case group was 0.61 ± 0.08 and in control group was 0.58 ± 0.08, which was significantly different. It has been reported in some previous studies that AIP value between 0.1–0.24 shows medium cardiac risk (62). Wu et al. were reported that AIP was an independent predictor of CAD (63). Compared to LDL-C or TC, AIP has been found to be a more helpful indicator of atherogenicity and CVD risk (64). Because vitamin D signaling reduces the expression of TNF-alpha, IL-6, IL-1, and IL-8 in isolated blood monocytes, it may have an impact on the pathogenesis of atherosclerosis (65, 66). By increasing nuclear factor kappa B (NF-κB) activation, vitamin D insufficiency was found to hasten the development of coronary artery disease in pigs, thereby demonstrating the anti-inflammatory properties of vitamin D (67). A characteristic of the development of atherosclerosis is the creation of foam cells originating from macrophages (68). It has been reported in previous studies that vitamin D can decrease cholesterol accumulation in macrophages and LDL uptake in Atheromas (69). Additionally, it alters the expression of tissue factor and thrombomodulin in monocytes, which has an impact on platelet aggregation and thrombogenic activity (70). In the Nakagawa et al. study, 1,25(OH)2D decreased matrix metalloproteinase (MMP)-2 and MMP-9 expression in cell culture, potentially avoiding plaque instability, luminal rupture, and thrombosis (71). Furthermore, 1,25(OH)2D inhibited foam cell production in macrophages isolated from patients with hypertension, diabetes, and obesity (72).

We also found that serum 25(OH)D levels showed significant inverse correlations with serum RBP-4 and positive correlation with omentin-1. In line with our findings, Dikker et al. found that omentin concentration had a positive correlation with vitamin D levels among the postmenopausal women (73). Also, Nazar et al. in another case control study found a linear correlation between vitamin D status and omentin-1 levels and also reported that vitamin D and omentin-1 deficiency maybe increase the risk of coronary artery disease (74). In contrast with these findings, Zorlu et al. in a cross-sectional study among the 77 female volunteers were reported that Omentin levels correlated significantly and negatively with the vitamin D (25). Omentin-1 is a 34-kDa, anti-inflammatory, circulating adipocytokine, has been considered to have a significant role in endothelial dysfunction, atherosclerosis and myocardial remodeling (75). It has been reported that omentin-1 exert its anti-inflammatory effects by suppressing some of the cytokines cascades and factors such as TNF-alpha. Also, omentin-1 induce the production of activated B cells in endothelial cells *via* nuclear factor kappa-light-chain. Moreover, the protein kinase (5’AMP) that activated by Omentin-1 can suppress the expression of vascular adhesion molecule E-selectin (74). Many researchers found that the serum levels of RBP-4 are associated with risk of metabolic syndrome (76, 77). Jialal and cols. reported that serum RBP-4 concentration would be independent predictors of CVD in diabetes (78).

Based on our knowledge, the present study is the first study that evaluated the association between vitamin D status with several cardiometabolic factors among the patients with MetS. The current study had several strengths, including the evaluation of several biochemical factors related to the risk of cardiovascular diseases, strong methodology and controlling the covariates between case and control. However, there were some limitations in the present study that should be considered in interpreting the results. Firstly, because of the study design, the cause-and-effect association will not be possible. Second, although the sample size in this study is 195, some potential associations between vitamin D and individual biomarkers of MetS might have not surfaced. Higher sample size might have discovered some additional relationships between vitamin D and markers of MetS.

## Conclusion

The results of the present study showed that participants with vitamin D deficiency had higher concentration of some MetS and CVD related biomarkers. Also, we found a significant association between 25(OH)D status and some of the adipokines and atherogenic indices in patients with MetS. Given the widespread vitamin D deficiency among the Iranian participants specially women, it has been suggested that fortification of some staple foods such as vegetable oils or cereals and dairy products with this vitamin can be appropriate therapeutic strategies to improve the vitamin D status in society. More studies with a higher sample size, especially clinical trials with strong methodology, are needed to re-evaluate the effect of vitamin D supplementation on the factors investigated in this study.

## Data availability statement

The raw data supporting the conclusions of this article will be made available by the authors, without undue reservation.

## Ethics statement

The studies involving human participants were reviewed and approved by Ethics Committee of Zabol University of Medical Sciences. The patients/participants provided their written informed consent to participate in this study.

## Author contributions

FA, MR, and ZK designed the study and analyzed data. SH-S, FA, and ZK cooperated in the implementation of the study. All authors contributed to the article and approved the submitted version.
